# Influence of Personal Protective Equipment on the Quality of Chest Compressions: A Meta-Analysis of Randomized Controlled Trials

**DOI:** 10.3389/fmed.2021.733724

**Published:** 2021-11-26

**Authors:** Ying Cui, Siyi Jiang

**Affiliations:** Intensive Care Unit, The Fourth Affiliated Hospital Zhejiang University School of Medicine, Yiwu, China

**Keywords:** chest compressions, personal protective equipment, cardiopulmonary resuscitation, meta-analysis, randomized controlled trial, simulation studies

## Abstract

**Background:** Randomized controlled trials (RCTs) evaluating the influence of personal protective equipment (PPE) on quality of chest compressions during cardiopulmonary resuscitation (CPR) showed inconsistent results. Accordingly, a meta-analysis was performed to provide an overview.

**Methods:** Relevant studies were obtained by search of Medline, Embase, and Cochrane's Library databases. A random-effect model incorporating the potential heterogeneity was used to pool the results.

**Results:** Six simulation-based RCTs were included. Overall, pooled results showed that there was no statistically significant difference between the rate [mean difference (MD): −1.70 time/min, 95% confidence interval (CI): −5.77 to 2.36, *P* = 0.41, *I*^2^ = 80%] or the depth [MD: −1.84 mm, 95% CI: −3.93 to 0.24, *P* = 0.11, *I*^2^ = 73%] of chest compressions performed by medical personnel with and without PPE. Subgroup analyses showed that use of PPE was associated with reduced rate of chest compressions in studies before COVID-19 (MD: −7.02 time/min, 95% CI: −10.46 to −3.57, *P* < 0.001), but not in studies after COVID-19 (MD: 0.14 time/min, 95% CI: −5.77 to 2.36, *P* = 0.95). In addition, PPE was not associated with significantly reduced depth of chest compressions in studies before (MD: −3.34 mm, 95% CI: −10.29 to −3.62, *P* = 0.35) or after (MD: −0.97 mm, 95% CI: −2.62 to 0.68, *P* = 0.25) COVID-19. No significant difference was found between parallel-group and crossover RCTs (*P* for subgroup difference both > 0.05).

**Conclusions:** Evidence from simulation-based RCTs showed that use of PPE was not associated with reduced rate or depth of chest compressions in CPR.

## Introduction

High-quality cardiopulmonary resuscitation (CPR) is the most important life-saving procedure for people with critical clinical conditions, such as cardiac arrest (1–3). The success of CPR relies on effective chest compression, which is defined as compressions with adequate rate, depth, and minimized interruptions by the International Liaison Committee on Resuscitation (4). With the pandemic of coronavirus disease 2019 (COVID-19), increasing number of critically ill patients with COVID-19 need CPR (5). Since there is evidence for COVID-19 infection transmission during chest compressions, use of personal protective equipment (PPE) has been recommended for health care personnel involved in resuscitating of patients with confirmed or suspected COVID-19 (6–8). However, studies evaluating the influence of PPE use on the quality of chest compression showed inconsistent results (9–14). Some simulation-based randomized controlled trials (RCTs) suggested that use of PPE by the CPR providers was associated with compromised quality of chest compression (9, 14), while others did not show significant difference regarding the performance of chest compression (10–13). An early meta-analysis including five studies (three RCTs and two observational studies) supported that the use of PPE may compromise the quality of chest compression during CPR (15). However, including observational studies may confound the results (15). Since three additional RCTs have been published since the meta-analysis (11–13), we performed an updated meta-analysis to provide an overview of current understanding regarding the influence of PPE use on quality of chest compression during the process of CPR.

## Methods

The PRISMA (Preferred Reporting Items for Systematic Reviews and Meta-Analyses) statement (16) and the Cochrane Handbook guidelines (17) were followed during the designing and implementation of the study.

### Search Strategy

Medline, Embase, and the Cochrane Library (Cochrane Center Register of Controlled Trials) databases were searched for relevant studies with a combined strategy as [(“personal protective equipment” OR “PPE”) AND (“chest compression” OR “cardiopulmonary resuscitation” OR “CPR”)]. Animal studies were not considered. The references of related reviews and original articles were also searched as a complementation. The final database search was conducted on May 12, 2021.

### Study Selection

Studies that fulfilled the following criteria were included: (1) studies published as full-length articles in English; (2) designed as simulation-based RCTs, either parallel group or crossover; (3) included medical personnel who were qualified to perform CPR and randomly allocated to perform chest compression with and without the use of PPE; (4) only adult CPR studies were included; and (5) outcome regarding the quality of chest compression was reported, including the difference of rate and/or the depth of chest compression between the two groups. Reviews, editorials, study protocols, observational studies, and studies performed in pediatric settings were excluded.

### Data Extraction and Quality Assessment

Database search, data extraction, and quality evaluation were conducted by two independent authors. If disagreement occurred, it was resolved by consensus. We extracted data regarding study information (first author, publication year, and study country), study design (blind or open-label), characteristics of medical personnel involved [sample size, age, sex, and body mass index (BMI)], details of PPE used, and durations for each cycle of chest compression. Quality evaluation was achieved using the Cochrane's Risk of Bias Tool (17) according to the following aspects: (1) random sequence generation; (2) allocation concealment; (3) blinding of participants and personnel; (4) blinding of outcome assessors; (5) incomplete outcome data; (6) selective outcome reporting; and (7) other potential bias.

### Statistical Analysis

Differences of rate and/or the depth of chest compression between participants with and without the use of PPE were separately evaluated via mean difference (MD) and their 95% confidence interval (CI) in this meta-analysis. We used the Cochrane's *Q*-test to detect the heterogeneity (18). The *I*^2^-statistic was also calculated, and an *I*^2^ > 50% reflected significant heterogeneity. Pooled analyses were calculated using a random-effect model because this method incorporates the influence of potential heterogeneity and retrieves a more generalized result (17). Sensitivity analysis by excluding one study at a time was used to evaluate the influence of each study on the pooled results of the meta-analysis (17). Subgroup analyses were performed to evaluate the results in studies published before or after the occurrence of COVID-19 and according to the design of the studies. We performed subgroup analysis according to the timing mainly because studies published after COVID-19 are more likely to be designed for the chest compression under the circumstances of possible COVID-19 positive patients, which may be more meaningful considering the current pandemic of COVID-19. Publication bias was evaluated by visual inspection of funnel plots, and the Egger's regression asymmetry test (19). *P*-values < 0.05 were considered statistically significant. The RevMan (Version 5.1; Cochrane, Oxford, UK) and Stata software (Version 12.0; Stata, College Station, TX) were applied for statistical analyses.

## Results

### Search Results

**Figure 1:**
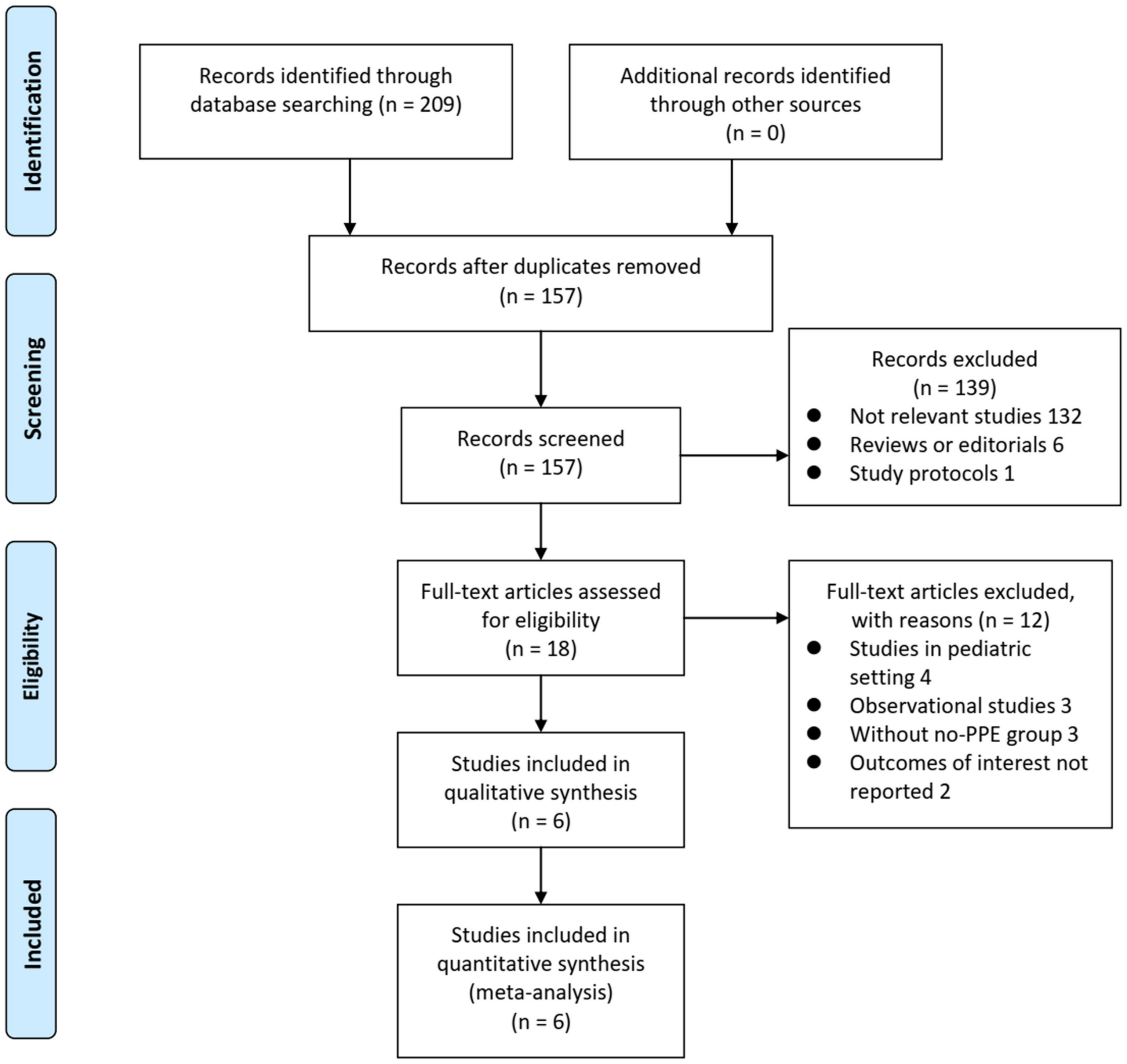
Flowchart of literature search.

The process of database search and study identification was shown in Figure 1. Briefly, 209 articles were obtained through the initial database search, and 157 were retrieved after exclusion of duplicated records. Among them, 139 articles were subsequently excluded based on titles and abstracts primarily because these studies were irrelevant to the aim of the meta-analysis. Of the 18 articles that underwent full-text review, 12 were further excluded for the reasons presented in Figure 1 Finally, six RCTs (9–14) were included.

### Study Characteristics

Table 1 shows the characteristics of the included studies. Overall, these studies were all simulation-based RCTs including medical personnel who performed chest compression on a manikin with and without the use of PPE. Two of the studies were performed before the occurrence of COVID-19 (9, 10), while the other four were performed after COVID-19 (11–14). These RCTs were performed in China (9, 14), Korea (10), Italy (12, 13), and Austria (11). Four of them were randomized crossover studies (9–11, 13), while the remaining two were randomized parallel-group studies (12, 14). A total of 258 medical personnel who were qualified to perform CPR were involved in the included studies (mean age: 31.3 years, men: 60%). Level-C PPE was used in five studies (9–13), and N95 mask was used in the other study (14). The level-C PPE applied among the included studies typically included safety gloves, safety glasses, chemical protective suits, respirator masks with active filter, and safety gumboots, etc., which have been specified for each study in Table 1. The duration of chest compression was 2 min in five studies (9, 11–14), and 4 min in the other study (10). Outcomes of rate and depth of chest compression between those with and without the use of PPE were reported in all of the included studies.

**Table 1:** Characteristics of the included RCTs.

**Study**	**Country**	**Design**	**Participants**	**Sample size**	**Mean age (years)**	**Male (%)**	**Mean BMI (kg/m^**2**^)**	**PPE used**	**Duration (min)**	**Quality score**
Chen et al. (9)	China	R, CO	Anesthesia residents	40	27.3	50.0	21.6	Safety gloves, chemical protective clothing, a respirator mask with active filter, and safety gumboots	2	4
Kim et al. (10)	Korea	R, CO	Emergency medical technicians	20	33.4	60.0	NR	A level-C PPE and a full face-piece reusable respirator with chemical-resistant gloves and boots	4	3
Tian et al. (14)	China	R, PG	Physicians and nurses	80	31.5	46.3	21.7	N95 mask	2	4
Raunch et al. (13)	Italy	R, CO	Emergency medical technicians	34	40.0	62.0	24.3	FFP3 mask, safety glasses, gloves, and a long-sleeved gown	2	4
Kienbacher et al. (11)	Austria	R, CO	Medical service providers	48	28.0	92.0	NR	A jumpsuit, safety glasses, latex gloves, and FFP2 mask with or without an expiration valve	2	3
Mormando et al. (12)	Italy	R, PG	Emergency medicine and anesthesiology senior residents	36	30.0	58.3	NR	Chemical resistant clothing, a full visor mask connected to an filter, and well-fitting, nonsterile gloves	2	5

*RCT, randomized controlled trials; BMI, body mass index; PPE, personal protection equipment; FFP, filtering face piece; R, randomized; CO, crossover; PG, parallel group; NR, not reported*.

### Data Quality

Table 2 shows the details of study quality evaluation. All of the included RCTs were open-label studies. Methods of random sequence generation were reported in four RCTs (9, 12–14), and information of allocation concealment was reported in one study (12). The overall quality score varied between 3 and 5, indicating moderate study quality.

**Table 2:** Details of quality evaluation for the included RCTs via the Cochrane's Risk of Bias Tool.

**Study**	**Random sequence generation**	**Allocation concealment**	**Blinding in performance**	**Blinding in outcome detection**	**Incomplete outcome data**	**Reporting bias**	**Other bias**	**Total**
Chen et al. (9)	Low	Unclear	High	High	Low	Low	Low	4
Kim et al. (10)	Unclear	Unclear	High	High	Low	Low	Low	3
Tian et al. (14)	Low	Unclear	High	High	Low	Low	Low	4
Raunch et al. (13)	Low	Unclear	High	High	Low	Low	Low	4
Kienbacher et al. (11)	Unclear	Unclear	High	High	Low	Low	Low	3
Mormando et al. (12)	Low	Low	High	High	Low	Low	Low	5

*RCTs, randomized controlled trials*.

### Meta-Analysis Results

**Figure 2:**
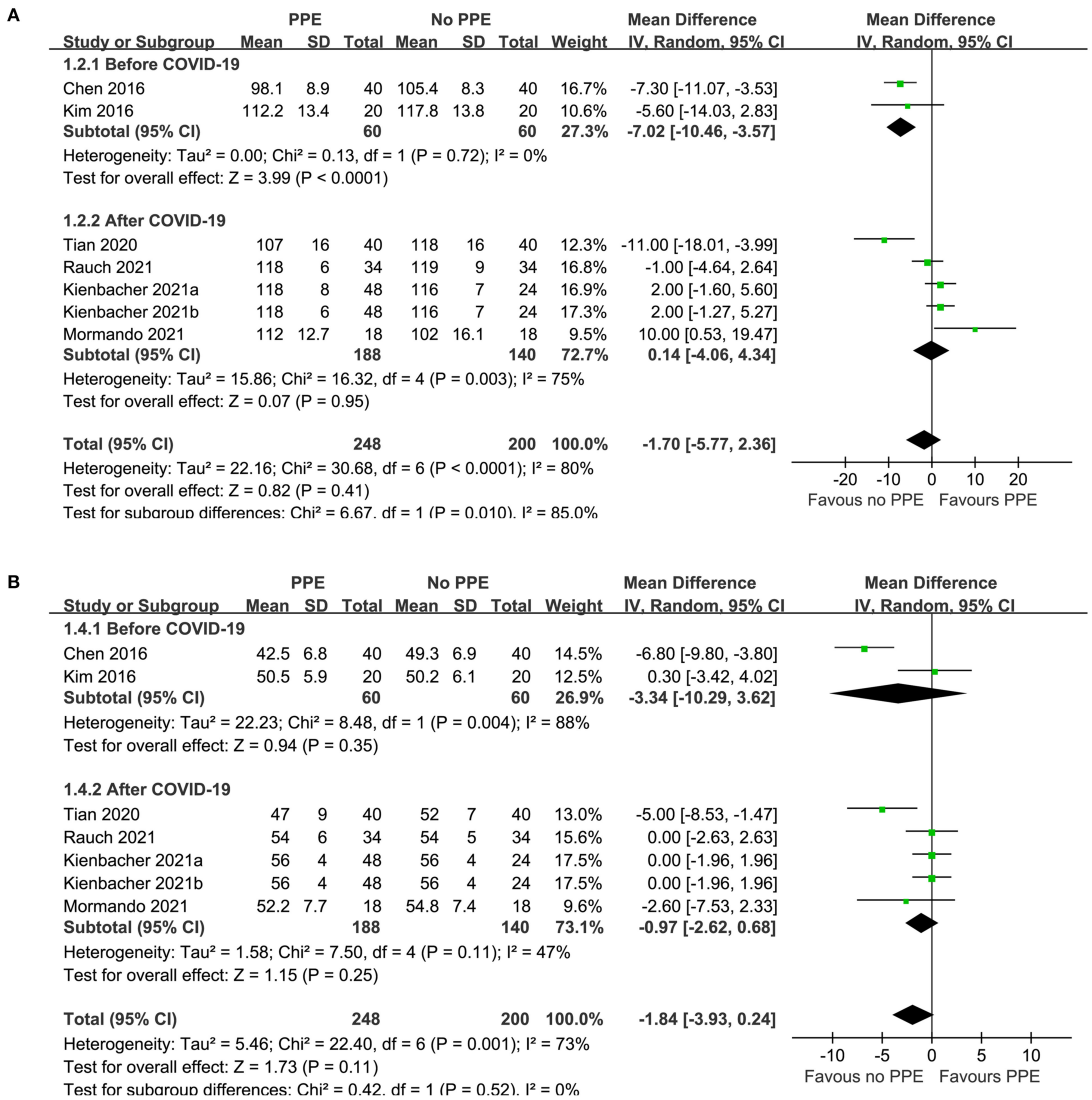
Forest plots for the meta-analysis of the quality of chest compression in medical personnel with and without PPE. **(A)** Forest plots for the meta-analysis of the rate of chest compression in studies before or after COVID-19 and **(B)** forest plots for the meta-analysis the depth of chest compression in studies before or after COVID-19.

**Figure 3:**
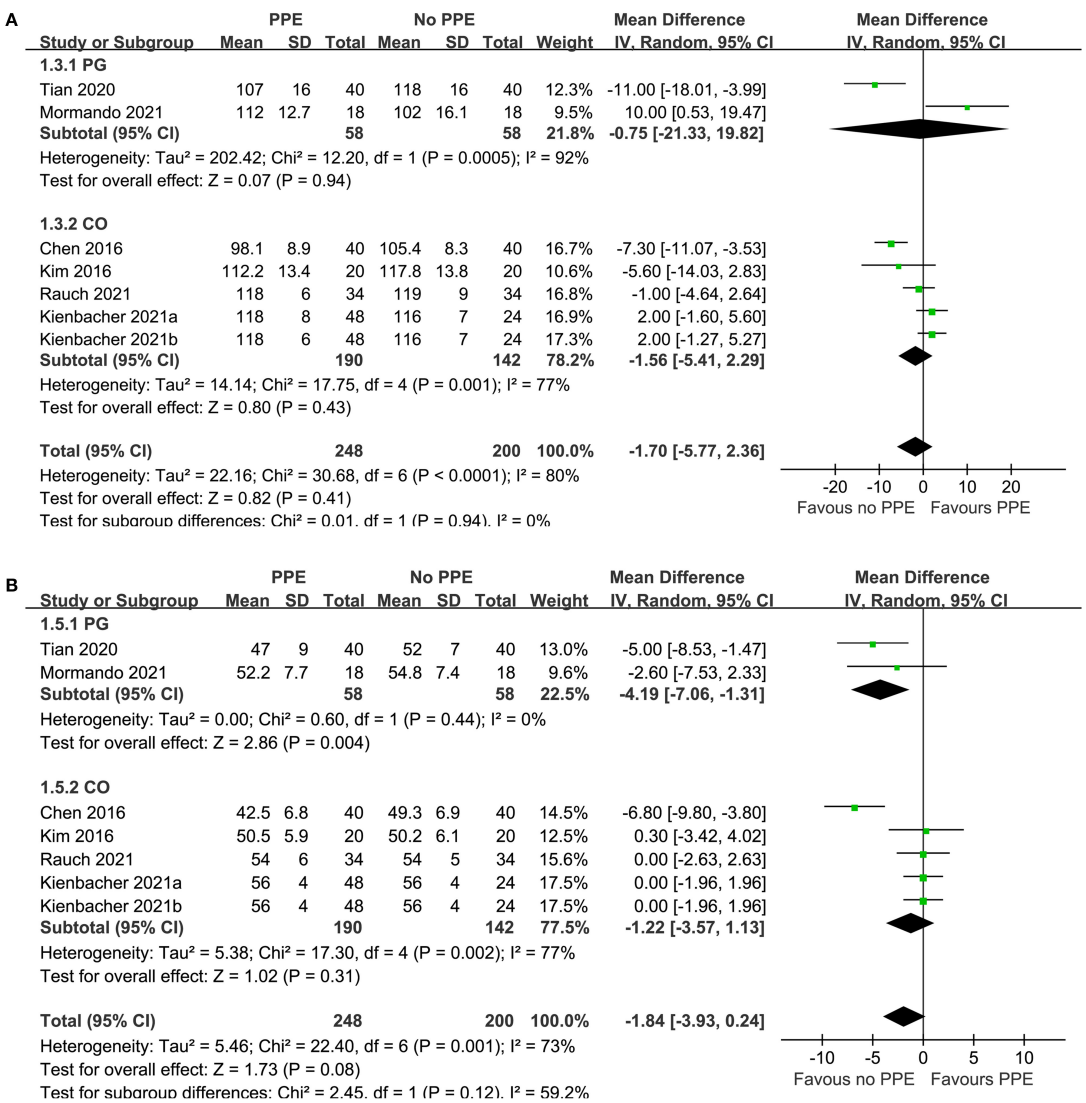
Forest plots for the meta-analysis of the quality of chest compression in medical personnel with and without PPE. **(A)** Forest plots for the meta-analysis of the rate of chest compression in parallel group (PG) or crossover (CO) RCTs and **(B)** forest plots for the meta-analysis the depth of chest compression in parallel group PG or CO RCTs.

Since one study (11) reported data of caregivers who provided CPR with two different sets of PPE, PPE including a filtering face piece (FFP) 2 mask with valve, and with PPE including an FFP2 mask without valve, these datasets were independently included into the meta-analysis. Overall, pooled results of seven datasets from six RCTs showed that there was no significant difference in the rate (MD: −1.70 time/min, 95% CI: −5.77 to 2.36, *P* = 0.41, *I*^2^ = 80%; Figure 2A) or the depth (MD: −1.84 mm, 95% CI: −3.93 to 0.24, *P* = 0.11, *I*^2^ = 73%; Figure 2B) of chest compression between medical personnel with and without PPE. Sensitivity analyses by excluding one dataset at a time did not significantly change the results (MD for rate of chest compression: −2.86 to −0.45, *P* all > 0.05; MD for depth of chest compression: −2.26 to −0.76, *P* all > 0.05; Table 3). Specifically, sensitivity analysis limited to studies with level-C PPE (9–13) showed consistent result (chest compression rate: MD: −0.45 time/min, 95% CI: −4.40 to 3.51, *P* = 0.83; *I*^2^ = 78%; chest compression depth: MD: −1.36 mm, 95% CI: 3.49 to 0.77, *P* = 0.21; *I*^2^ = 72%; Table 3). Subgroup analyses showed that use of PPE was associated with reduced rate of chest compression in studies before COVID-19 (MD: −7.02 time/min, 95% CI: −10.46 to −3.57, *P* < 0.001), but not in studies after COVID-19 (MD: 0.14 time/min, 95% CI: −5.77 to 2.36, *P* = 0.95; Figure 2A). In addition, PPE was not associated with a significantly reduced depth of chest compression in studies before (MD: −3.34 mm, 95% CI: −10.29 to −3.62, *P* = 0.35) or after (MD: −0.97 mm, 95% CI: −2.62 to 0.68, *P* = 0.25; Figure 2B) the occurrence of COVID-19. Moreover, subgroup analyses according to study design were shown in Figures 3A,B, which showed no significant between-subgroup differences of the rate or the depth of chest compression in parallel-group and crossover studies (both *P* for subgroup difference > 0.05).

**Table 3:** Sensitivity analyses.

**Dataset excluded**	**MD (95% CI)**	***I*** **^2^ (%)**	***P*** **for Cochrane's *Q*-test**	***P*** **for overall effect**
**Compression Rate (time/min)**				
Chen et al. (9)	−0.54 [−4.47, 3.40]	73	0.003	0.79
Kim et al. (10)	−1.24 [−5.63, 3.16]	83	<0.001	0.58
Tian et al. (14)	−0.45 [−4.40, 3.51]	78	<0.001	0.83
Raunch et al. (13)	−1.84 [−6.92, 3.25]	84	<0.001	0.48
Kienbacher et al. (11)	−2.44 [−7.21, 2.34]	81	<0.001	0.32
Kienbacher et al. (11)	−2.46 [−7.24, 2.32]	81	<0.001	0.31
Mormando et al. (12)	−2.86 [−6.88, 1.15]	81	<0.001	0.16
**Compression Depth (mm)**
Chen et al. (9)	−0.76 [−2.19, 0.66]	35	0.17	0.29
Kim et al. (10)	−2.17 [−4.50, 0.16]	77	<0.001	0.07
Tian et al. (14)	−1.36 [−3.49, 0.77]	72	0.003	0.21
Raunch et al. (13)	−2.21 [−4.66, 0.24]	77	<0.001	0.08
Kienbacher et al. (11)	−2.26 [−4.79, 0.27]	75	<0.001	0.08
Kienbacher et al. (11)	−2.26 [−4.79, 0.27]	75	<0.001	0.08
Mormando et al. (12)	−1.78 [−4.05, 0.50]	77	<0.001	0.13

*MD, mean difference; CI, confidence interval*.

### Publication Bias

**Figure 4:**
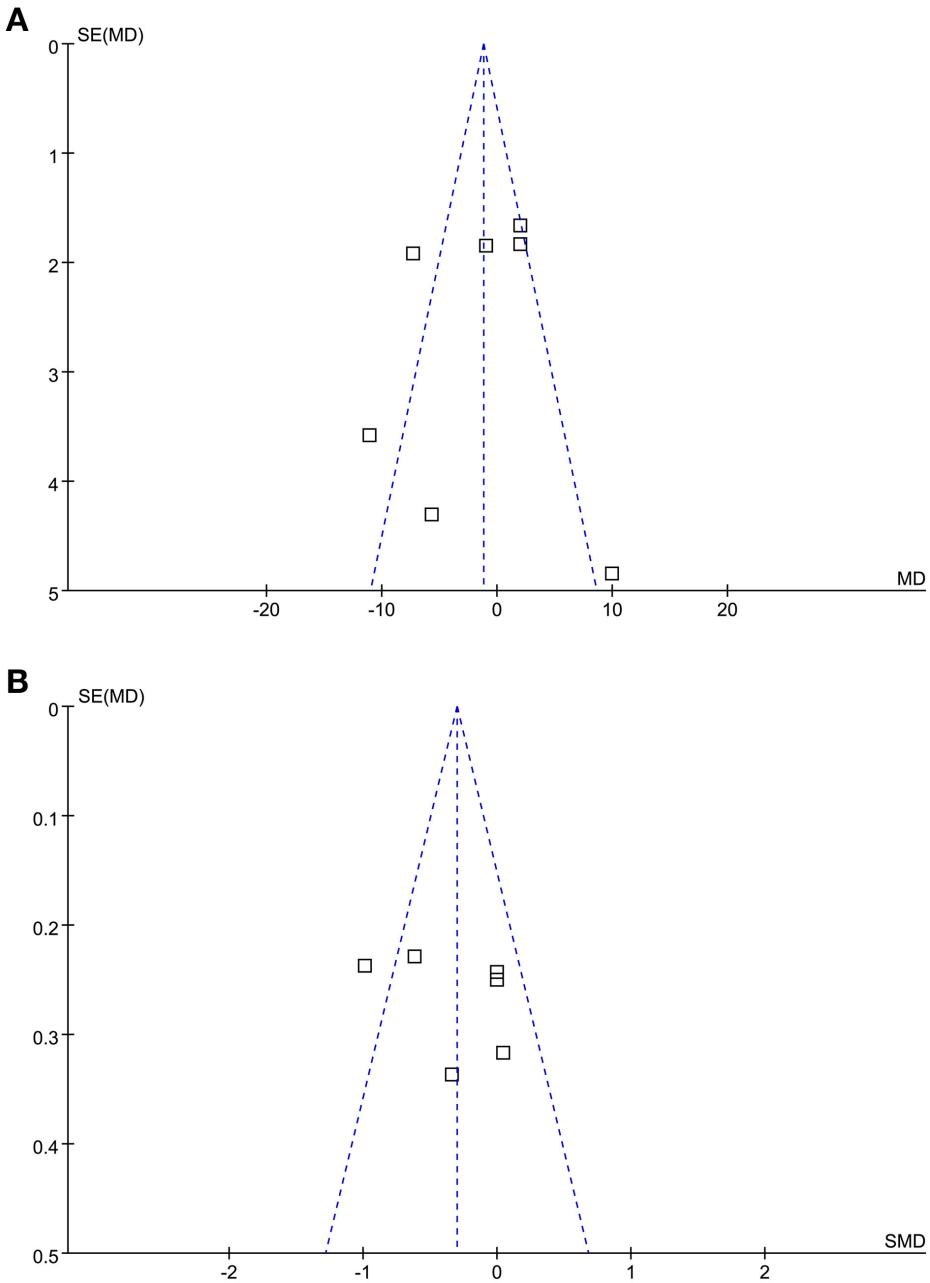
Funnel plots for the meta-analysis of the quality of chest compression in medical personnel with and without PPE. **(A)** Funnel plots for the outcome of the rate of chest compression and **(B)** funnel plots for the outcome of the depth of chest compression.

The funnel plots for the meta-analysis of the rate and depth of chest compression between medical personnel with and without PPE were shown in Figures 4A,B. These funnel plots were symmetrical on visual inspection, suggesting low risk of publication biases. Egger's regression tests were not performed since only seven datasets were available for each outcome.

## Discussion

In this meta-analysis, by pooling the results of up-to-date simulation-based RCTs, we found that use of PPE was not associated with reduced rate or depth of chest compression in CPR. The robustness of the finding was confirmed by results of sensitivity analysis by excluding one dataset at a time. Moreover, subgroup analysis showed consistent results for RCTs published after the occurrence of COVID-19. Taken together, these results suggest that PPE use in providers of CPR dose not compromise the quality of chest compression. Since there is evidence for COVID-19 infection transmission during chest compressions (6), results of this meta-analysis further supported the current recommendation that PPE should be worn during chest compressions for patients with confirmed or suspected COVID-19.

Results of our meta-analysis are different from the previous meta-analysis which showed that use of PPE may compromise the quality of chest compression during CPR (15). Some difference in methodology of meta-analysis should be noticed when the results of current meta-analysis and the previous one are compared. Firstly and the most importantly, although all of the included studies were simulation-based, the previous meta-analysis included both RCTs and observational studies (before and after PPE). Including the non-randomized before-and-after-PPE studies may confound the results of the overall meta-analysis since the procedures of chest compression with PPE were all performed after a few sessions without PPE (15). In our meta-analysis, only RCTs were included. Although four of the RCT included in our meta-analysis were crossover studies (9–11, 13), the sequences for performing chest compression with or without PPE were randomly allocated, which had minimized influence on the results. In addition, the previous meta-analysis included one study in the pediatric setting (15). Since the delivering of chest compressions to adult and pediatric patients is different, including study in the pediatric setting may also cause additional bias. Unlike the previous one, only RCTs of chest compressions performed in adult patients setting were included in our meta-analysis. Finally, only one out of the five included studies in the previous meta-analysis was performed during the COVID-19 pandemic (15). In our meta-analysis four of the six included RCTs (11–14) were performed during the COVID-19 pandemic, and further subgroup analysis by including these four studies only also showed that use of PPE did not reduce the rate or depth of chest compressions in CPR. This is important because PPE used in these studies generally mimicked the PPE used in chest compressions for patients with suspected or confirmed COVID-19. Collectively, results of our meta-analysis suggest that use of PPE was not associated with reduced rate or depth of chest compressions in CPR, and the results were consistent for including studies during the COVID-19 pandemic.

Evidence for optimal PPE during CPR is limited. One included RCT showed that PPEs including masks with and without expiration valve were both safe for use during CPR and did not significantly affect the quality of chest compressions (11). However, another non-randomized study showed that protective masks other than surgical masks used as PPE increase rescuer fatigue in CPR and negatively affect the quality of chest compressions (20). However, due to the non-randomized nature and lack of a control group without PPE, these results are difficult to interpret. Besides, automated chest compression devices and reduced duration of the cycle of CPR have also been suggested for patients with suspected or confirmed COVID-19 during CPR (21, 22). The optimal PPE may be that has minimized influence on the quality of chest compression, but has adequacy protection for the healthcare providers. More researches are needed for the development of an evidence-based CPR guideline for patients with COVID-19.

Our study has limitations. Firstly, the search strategy of this study was based on keywords, rather than using MeSH (Medline) and Emtree (EMBASE) as instructed by the Cochrane Handbook (17), and a librarian was not consulted during the development of the search strategy. Accordingly, search strategy in this study is not sufficient for a robust search. Besides, all of the included RCTs were simulation-based, and could not fully reflect the situation of real-life scenarios such as CPR for critically ill patients with COVID-19. In addition, the number of available studies and participants in each study are limited, which prevented comprehensive subgroup analysis according to the characteristics of the CPR providers and studies. Moreover, rate and depth were used to evaluate the quality of chest compressions during CPR. It remains unknown whether use of PPE may affect the clinical outcomes in patients receiving CPR. Finally, significant heterogeneity was observed among the included studies. Although we found consistent results for studies during COVID-19 pandemic, future studies are needed to determine whether the difference in PPE may affect the results.

In conclusion, results of the meta-analysis including simulation-based RCTs showed that use of PPE was not associated with reduced rate or depth of chest compressions in CPR. These findings support the current recommendation that PPE should be worn during chest compressions for patients with confirmed or suspected COVID-19.

## Data Availability Statement

The original contributions presented in the study are included in the article/supplementary material, further inquiries can be directed to the corresponding author/s.

## Author Contributions

YC and SJ conceived and designed the study, performed database search, study identification, data extraction, statistical analyses, and results interpretation. YC drafted the manuscript. Both authors revised the manuscript and approved the submission.

## Conflict of Interest

The authors declare that the research was conducted in the absence of any commercial or financial relationships that could be construed as a potential conflict of interest.

## Publisher's Note

All claims expressed in this article are solely those of the authors and do not necessarily represent those of their affiliated organizations, or those of the publisher, the editors and the reviewers. Any product that may be evaluated in this article, or claim that may be made by its manufacturer, is not guaranteed or endorsed by the publisher.
